# Homeopathy with a Whiskey-Cure Annex

**Published:** 1892-04

**Authors:** 


### HOMŒOPATHY WITH A WHISKY-CURE ANNEX—A ROW IN THE CAMP.

"When thieves fall out, honest men get their deserts." "Bragg is a good dog, holdfast is a better."

There is a row in the homœopathic camp of Texas. An ex-President of the Homœopathic Medical Society, Dr. T. H. Bragg, of Austin, has taken on an annex to his practice, in the shape of a Keely-Institute — bichloride-of-brass — specific for drink-so-much-ia, chewing tobacco and cussin'; and Fisher, editor of the *Homœopathic Slogan*, the mouth-piece of homœopathic nonsense; ex-President of the Homœopathic Society, and Poo-bah-general of the clans, objects to it. He is out in a two-column wail over the defection of Bro. Bragg,—the tears being mingled with considerable gall and resentment, and the usual amount of crowing. Fisher has "had it in" for Bro. Bragg a long time, and now his opportunity presents, he makes the most of it.

It seems, a while back, Bragg was President of their little thirty-strong "State Homœopathic Association," and Fisher wanted them to adopt his journal as the official organ. Bragg opposed it, saying the homœopathy of the editor was not pure homœopathy; it was not the kind of homœopathy that layout wanted, it was not a fine enough piece of goods; in fact, intimated very strongly (we blush to say it), that it had "flies on it." And this defeated the official-organ-scheme.

Fisher and Bragg were partners here in Austin many years. We never knew till now that there was any difference between them, or between the kind of homœopathy they practiced. We have known a long time that they both practice almost any thing that comes handy,—allo-homo-hydro, or any other kind of pathy, and whenever they got hold of a serious case, called on a regular, generally, to help them out. But there was a difference, it seems, and Fisher has enlightened us somewhat. Their respective homœopathies were "*similia*,"—well, perhaps, even "similibus;" but there was no "curantur" in Bragg's homœopathy, and now because he proposes to add it in the shape of a whiskey-cur(e)antur, Fisher kicks, and says homœopathy and whiskey don't, won't, nor shouldn't mix; and asks what kind of homœopathy Bragg calls that? No flies on that style of homœopathy, eh? If Fisher's homœopathy was not a fine enough article for the brethren, he asks how they like the improved kind?

Fisher bewails the apostacy of Bragg, and says he has thrown the homœopathic adipose upon the glowing embers, so to speak; he has forever destroyed their hopes, he says, of State recognition (too bad); and of their ever getting control of one or all of the asylums in Texas (pity); has forever closed the doors to preferment to a chair in the Texas Medical College, etc., etc. All these blissful hopes, he says, are dashed by this act of Bragg's, which has indeed brought reproach upon even the name of homœopathy. He will probably be immolated upon the altar of homœopathic wrath at the next meeting of their society; unless he should be as lucky as a certain other ex-President (of "hepatic-pill" notoriety), and be "exonerated."

Another homœopath says of it, that Bragg went into it to make money, and as he is doing very little practice, homœopathic or any other kind, the whiskey cure will occupy his attention in the future; that in fact it will be, or is, a case where the tail will wag the dog, and not the dog the tail.

